# The unrestricted global effort to complete the COOL trial

**DOI:** 10.1186/s13017-023-00500-z

**Published:** 2023-05-11

**Authors:** Andrew W. Kirkpatrick, Federico Coccolini, Matti Tolonen, Samuel Minor, Fausto Catena, Emanuel Gois, Christopher J. Doig, Michael D. Hill, Luca Ansaloni, Massimo Chiarugi, Dario Tartaglia, Orestis Ioannidis, Michael Sugrue, Elif Colak, S. Morad Hameed, Hanna Lampela, Vanni Agnoletti, Jessica L. McKee, Naisan Garraway, Massimo Sartelli, Chad G. Ball, Neil G. Parry, Kelly Voght, Lisa Julien, Jenna Kroeker, Derek J. Roberts, Peter Faris, Corina Tiruta, Ernest E. Moore, Lee Anne Ammons, Elissavet Anestiadou, Cino Bendinelli, Konstantinos Bouliaris, Rosemarry Carroll, Marco Ceresoli, Francesco Favi, Angela Gurrado, Joao Rezende-Neto, Arda Isik, Camilla Cremonini, Silivia Strambi, Georgios Koukoulis, Mario Testini, Sandy Trpcic, Alessandro Pasculli, Erika Picariello, Fikri Abu-Zidan, Ademola Adeyeye, Goran Augustin, Felipe Alconchel, Yuksel Altinel, Luz Adriana Hernandez Amin, José Manuel Aranda-Narváez, Oussama Baraket, Walter L. Biffl, Gian Luca Baiocchi, Luigi Bonavina, Giuseppe Brisinda, Luca Cardinali, Andrea Celotti, Mohamed Chaouch, Maria Chiarello, Gianluca Costa, Nicola de’Angelis, Nicolo De Manzini, Samir Delibegovic, Salomone Di Saverio, Belinda De Simone, Vincent Dubuisson, Pietro Fransvea, Gianluca Garulli, Alessio Giordano, Carlos Gomes, Firdaus Hayati, Jinjian Huang, Aini Fahriza Ibrahim, Tan Jih Huei, Ruhi Fadzlyana Jailani, Mansoor Khan, Alfonso Palmieri Luna, Manu L. N. G. Malbrain, Sanjay Marwah, Paul McBeth, Andrei Mihailescu, Alessia Morello, Francesk Mulita, Valentina Murzi, Ahmad Tarmizi Mohammad, Simran Parmar, Ajay Pak, Michael Pak-Kai Wong, Desire Pantalone, Mauro Podda, Caterina Puccioni, Kemal Rasa, Jianan Ren, Francesco Roscio, Antonio Gonzalez-Sanchez, Gabriele Sganga, Maximilian Scheiterle, Mihail Slavchev, Dmitry Smirnov, Lorenzo Tosi, Anand Trivedi, Jaime Andres Gonzalez Vega, Maciej Waledziak, Sofia Xenaki, Desmond Winter, Xiuwen Wu, Andee Dzulkarnean Zakaria, Zaidi Zakaria

**Affiliations:** 1 Departments of Surgery and Critical Care Medicine, University of Calgary, Foothills Medical Centre, Calgary, AB EG23T2N 2T9 Canada; 2 General, Emergency and Trauma Surgery Department, Pisa University Hospital, Pisa, Italy; 3 Abdominal Center, Helsinki University Hospital and University of Helsinki, Helsinki, Finland; 4 Departments of Critical Care Medicine and Surgery, Dalhousie University, Halifax, NS Canada; 5 Department of Surgery, Bufalini Hospital, Cesena, Italy; 6 Department of Surgery, Londrina State University, and National COOL Coordinator for Brazil, Londrina, Brazil; 7 Departments of Critical Care Medicine and Community Health Sciences, Cumming School of Medicine, University of Calgary, Calgary, AB Canada; 8 Department of Clinical Neuroscience and Hotchkiss Brain Institute, Cumming School of Medicine, University of Calgary and Foothills Medical Centre, Calgary, AB Canada; 9 General Surgery I, San Matteo Hospital Pavia, University of Pavia, Pavia, Italy; 10 Emergency Surgery and Trauma Center, University of Pisa, Pisa, Italy; 11 4th Department of Surgery, Medical School, Aristotle University of Thessaloniki, General Hospital “George Papanikolaou”, Thessaloniki, Greece; 12 Letterkenny University Hospital, Donegal, Ireland; 13 University of Samsun, Samsun Training and Research Hospital, Samsun, Turkey; 14 Department of Surgery, University of British Columbia, Vancouver, BC Canada; 15 Department of Gastroenterological Surgery, Helsinki University Hospital and University of Helsinki, Espoo, Finland; 16 Chief Anesthesiology, Bufalini Hospital, Cesena, Italy; 17 Global Project Manager, COOL Trial and the TeleMentored Ultrasound Supported Medical Interventions Research Group, Calgary, AB Canada; 18 Departments of Surgery and Critical Care Medicine, University of British Columbia, Vancouver, BC Canada; 19 Department of Surgery, Macerata Hospital, Global Alliance for Infections in Surgery, Macerata, Italy; 20 Trauma and Acute Care Surgery, Foothills Medical Center, Calgary, AB Canada; 21 Departments of Surgery and Medicine, Schulich School of Medicine and Dentistry, Western University, London, ON Canada; 22 Department of Surgery, NSHA-Queen Elizabeth II Health Sciences Center, Dalhousie University, Halifax, NS Canada; 23 Division of Vascular and Endovascular Surgery, Department of Surgery and School of Epidemiology and Public Health, Faculty of Medicine, University of Ottawa, Ottawa, ON Canada; 24 University of Calgary, Calgary, AB Canada; 25 Alberta Health Services, Calgary, AB Canada; 26 Ernest E. Moore Shock Trauma Center, University of Colorado, Denver, CO USA; 27 Department of Surgery, Denver Health, Denver, CO USA; 28 John Hunter Hospital, Newcastle, NSW Australia; 29 General Surgery Department of Koutlimbaneio, Triantafylleio General Hospital of Larissa, Larissa, Thessaly Greece; 30 General and Emergency Surgery, School of Medicine and Surgery, Milano-Bicocca University, Monza, Italy; 31 Chirurgia Generale E d’Urgenza, Ospedale M. Bufalini - Cesena, AUSL Della Romagna, Cesena, Italy; 32 Department of Precision and Regenerative Medicine and Ionian Area, Unit of Academic General Surgery “V. Bonomo”, University of Bari “A. Moro”, Bari, Italy; 33 Trauma and Acute Care Surgery, General Surgery, St. Michael’s Hospital, Toronto, ON Canada; 34 General Surgery Department, Istanbul Medeniyet University School of Medicine Istanbul, Istanbul, Turkey; 35 General Surgery Unit, Ospedale M. Buffalini Di Cesena, Cesena, Italy; 36 College of Medicine and Health Sciences, United Arab Emirates University, Al-Ain, United Arab Emirates; 37 Division of Surgical Oncology, Afe Babalola University Multisystem Hospital, Ado-Ekiti, Nigeria; 38 University Hospital Centre Zagreb, School of Medicine University of Zagreb, Zagreb, Croatia; 39 Virgen de la Arrixaca University Hospital IMIB-Arrixaca, Ctra. Madrid-Cartagena, S/N, Murcia, Spain; 40 Bagcilar Research and Training Hospital, Istanbul, Turkey; 41 Nurse Master of Nursing, Professor and Coordinator of the teaching-service relationship, Faculty of Health Sciences, University of Sucre, Sincelejo, Colombia; 42 Trauma and Emergency Surgery Unit. General, Digestive and Transplantation Surgery Department, University Regional Hospital of Málaga, Malaga, Spain; 43 Department of Surgery, Bizerte Hospital, Bizerte, Tunisia; 44 Scripps Clinic Medical Group, La Jolla, CA USA; 45 Department of Clinical and Experimental Sciences, University of Brescia, Brescia, Italy; 46 Department of Surgery, University of Milan Medical School, Milan, Italy; 47 Department of Surgery, Fondazione Policlinico Universitario A Gemelli IRCCS, Rome, Italy; 48 Department of Surgery, General Hospital Madonna del Soccorso, San Benedetto del Tronto, Italy; 49 General Surgery Unit, UO Chirurgia Generale - Ospedale Maggiore Di Cremona, Cremona, Italy; 50 Department of Visceral and Digestive Surgery, Monastir University, Monastir, Tunisia; 51 Department of Surgery, Azienda Sanitaria Provinciale Di Cosenza, Cosenza, Italy; 52 Fondazione Policlinico Campus Bio-Medico, University Campus Bio-Medico of Rome, Rome, Italy; 53 Colorectal and Digestive Surgery Unit–DIGEST Department, Beaujon University Hospital AP-HP, University Paris Cité, Clichy, France; 54 Department of General Surgery, Cattinara University Hospital, Trieste, Italy; 55 Department of Proctology, Clinic for Surgery, University Clinical Center Tuzla, Tuzla, Bosnia and Herzegovina; 56 Department of General Surgery, University of Insubria, University Hospital of Varese, ASST Sette Laghi, Regione Lombardia, Italy; 57 Unit of Digestive and Metabolic Minimally Invasive Surgery, Clinique Saint Louis, Poissy, Poissy, Ile de France, France; 58 Unit of Emergency and General Surgery, Guastalla Hospital, AUSL Reggio Emilia, Guastalla, Italy; 59 Chirurgie Digestive, Service de Chirurgie Vasculaire Et, Générale University Hospital of Bordeaux FR, Bordeaux, France; 60 Fondazione Policlinico A, Gemelli IRCCS, Rome, Italy; 61 Department Head of Surgery, Infermi Hospital, Rimini, Italy; 62 Emergency and General Consultant Surgeon, Nuovo Ospedale “S. Stefano”, Azienda ASL Toscana Centro, Prato, Italy; 63 Surgery Unit, Hospital Universitário Terezinha de Jesus, SUPREMA, Juiz de Fora, Brazil; 64 Department of Surgery, Faculty of Medicine and Health Sciences, Universiti Malaysia Sabah, Kota Kinabalu, Sabah, Malaysia; 65 Research Institute of General Surgery, Jinling Hospital, Medical School of Nanjing University, Nanjing, Jiangsu China; 66 Hospital Umum Sarawak/Universiti Malaysia, Sarawak, Malaysia; 67 Hospital Sultanah Aminah, Johor Bahru, Malaysia; 68 Universiti Sains Islam Malaysia, Nilai, Malaysia; 69 General Surgery, University Hospitals, Sussex, UK; 70 Universidad de Sucre, Medicine Program, Clínica Santa María, Sincelejo, Colombia; 71 First Department of Anaesthesiology and Intensive Therapy, Medical University of Lublin, Lublin, Poland; 72 International Fluid Academy, Lovenjoel, Belgium; 73 Postgraduate Institute of Medical Sciences, Rohtak, Haryana India; 74 University of Calgary, Calgary, Canada; 75 Tameside and Glossop Integrated Care NHSFT, Ashton-under-Lyne, UK; 76 Department of General Surgery, Madonna del Soccorso Hospital - San Benedetto del Tronto, Italy, Italy; 77 Department of Surgery, General University Hospital of Patras, Rio, Greece; 78 Department of Surgical Science, Cagliari State University, Cagliari, Italy; 79 Hospital Angkatan Tentera Tuanku Mizan, Kuala Lumpur, Malaysia; 80 Department of General Surgery, King George’s Medical University, Lucknow, UP India; 81 School of Medical Sciences & Hospital, Universiti Sains Malaysia, Kelantan, Malaysia; 82 University Hospital Careggi, Florence, Italy; 83 Department of Emergency Surgery, Cagliari University Hospital, Cagliari, Italy; 84 Fondazione Policlinico Universitario A. Gemelli IRCCS, Catholic University of Sacred Heart, Rome, Italy; 85 Department of General Surgery, Hüseyin Kemal Raşa, Anadolu Medical Center, Kocaeli, Turkey; 86 Division of General and Minimally Invasive Surgery, ASST Valle Olona, Busto Arsizio, Italy; 87 Emergency Surgery Unit and Trauma Team, Careggi University Hospital, Florence, Italy; 88 Plovdiv Medical University, Plovdiv, Bulgaria; 89 Department of Surgery, South Ural State Medical University, Chelyabinsk City, Russia; 90 Department of General Surgery, University of Bologna, Bologna, Italy; 91 Fiona Stanley Hospital, Perth, WA Australia; 92 Clinica Santa Maria, Sincelejo, Colombia; 93 Military Institute of Medicine, Warsaw, Poland; 94 Department of General Surgery, University Hospital of Heraklion, Crete, Greece; 95 St Vincent’s University Hospital, Dublin, Ireland; 96 Department of Surgery, School of Medical Sciences and Hospital USM, Universiti Sains Malaysia, Georgetown, Malaysia

**Keywords:** Intraperitoneal sepsis, Septic shock, Peritonitis, Open abdomen, Multiple organ dysfunction, Laparotomy, Randomized controlled trial, Global health

## Abstract

**Background:**

Severe complicated intra-abdominal sepsis (SCIAS) has an increasing incidence with mortality rates over 80% in some settings. Mortality typically results from disruption of the gastrointestinal tract, progressive and self-perpetuating bio-mediator generation, systemic inflammation, and multiple organ failure. A further therapeutic option may be open abdomen (OA) management with negative peritoneal pressure therapy (NPPT) to remove inflammatory ascites and attenuate the systemic damage from SCIAS, although there are definite risks of leaving the abdomen open whenever it might possibly be closed. This potential therapeutic paradigm is the rationale being assessed in the Closed Or Open after Laparotomy (COOL trial) (https://clinicaltrials.gov/ct2/show/NCT03163095). Initially, the COOL trial received Industry sponsorship; however, this funding mandated the use of a specific trademarked and expensive NPPT device in half of the patients allocated to the intervention (open) arm. In August 2022, the 3 M/Acelity Corporation without consultation but within the terms of the contract canceled the financial support of the trial. Although creating financial difficulty, there is now no restriction on specific NPPT devices and removing a cost-prohibitive intervention creates an opportunity to expand the COOL trial to a truly global basis. This document describes the evolution of the COOL trial, with a focus on future opportunities for global growth of the study.

**Methods:**

The COOL trial is the largest prospective randomized controlled trial examining the random allocation of SCIAS patients intra-operatively to either formal closure of the fascia or the use of the OA with an application of an NPPT dressing. Patients are eligible if they have free uncontained intraperitoneal contamination and physiologic derangements exemplified by septic shock OR severely adverse predicted clinical outcomes. The primary outcome is intended to definitively inform global practice by conclusively evaluating 90-day survival. Initial recruitment has been lower than hoped but satisfactory, and the COOL steering committee and trial investigators intend with increased global support to continue enrollment until recruitment ensures a definitive answer.

**Discussion:**

OA is mandated in many cases of SCIAS such as the risk of abdominal compartment syndrome associated with closure, or a planned second look as for example part of “damage control”; however, improved source control (locally and systemically) is the most uncertain indication for an OA. The COOL trial seeks to expand potential sites and proceed with the evaluation of NPPT agnostic to device, to properly examine the hypothesis that this treatment attenuates systemic damage and improves survival. This approach will not affect internal validity and should improve the external validity of any observed results of the intervention.

*Trial registration*: National Institutes of Health (https://clinicaltrials.gov/ct2/show/NCT03163095).

## Background

Sepsis is an increasing cause of death worldwide [2, 3], with an incidence estimated between 18 and 31 million cases worldwide per year [3–7]. Sepsis mortality approaches 30–40% when a shock is present [8-10], and may be higher in the developing world [2]. The incidence and mortality of sepsis can be compared to other critical global health problems such as COVID-19 with 6.5 million deaths worldwide over more than 2 years [11], or 4.4 million deaths from trauma each year [12]. Sepsis was the single most expensive medical condition in the USA in 2016, with 22.2 billion dollars spent just on in-hospital stays [13]. Intra-abdominal sepsis (IAS) is the 2nd most common form of sepsis, and may be particularly severe because of the unique anatomic, physiologic, and microbiologic characteristics of hollow viscera within the abdominal cavity [14]. IAS occurs within a semirigid anatomic container that is exquisitely affected by raised intra-compartmental pressure that quickly induces abdominal visceral malperfusion and ischemia [15, 16]. Further, the extensive flora of the human microbiome is contained within the abdominal container exacerbating any pathology in a multitude of ways that are yet only minimally understood [17, 18]. Thus, it has been reported that hospital mortality is highest for patients who have intra-abdominal infection secondary to ischemic bowel or disseminated infection [19].

Severe complicated intra-abdominal sepsis (SCIAS) represents a subset of IAS sepsis but is perhaps the most challenging clinical situation. Sartelli and the World Society of Emergency Surgery have defined IAS as severe when associated with organ dysfunction [9, 20–22], and as complicated when the inflammation or contamination spreads beyond a single organ, causing either localized or diffuse peritonitis [20, 23]. SCIAS may be distinguished from other causes of severe sepsis through a requirement for surgical abdominal exploration to address disruption in the gastrointestinal (GI) tract and provide source control.

Patients with SCIAS require early hemodynamic support, source control, and antimicrobial therapy [23]. Despite advances in diagnosis, surgery, and antimicrobial therapy, mortality rates associated with complicated intra-abdominal infections and IAS remain very high [22]. Failure to obtain adequate source control is often considered the driving cause of SCIAS and has been identified as an independent predictor of mortality [24]. Even with prompt appropriate therapy, SCIAS may progress to septic shock and multiple organ dysfunction, presumed as consequences of peritoneal and systemic inflammation. There is significant variability in the human immune response to an infectious focus, whereas some individuals produce a massive bio-mediator storm propagating multisystem organ failure and death, whereas other individuals may be anergic with little or no response to the same stimuli.

In patients with SCIAS, repeat laparotomy may be necessary to eliminate persistent peritonitis or new infectious foci [25–27]. Differentiating “failed source control” [28, 29] from a self-propagating bio-mediator storm is difficult or impossible without abdominal re-exploration. In a Dutch multicenter randomized controlled trial (RCT), 42% of those randomized to expectant management after laparotomy for IAS, underwent relaparotomy for suspected or proven persistent peritonitis [25]. Interestingly, 31% of the repeat laparotomies were negative. The results of the Dutch study concluded a previously long-standing debate concerning two closed surgical approaches to ensuring source control in the peritoneal cavity; that of “laparotomy on demand – (LOD)” versus “planned relaparotomy” (PRL) [25, 30, 31]. The relative merits of either approach were widely debated until the conduct of the above RCT [25]. Although this trial noted no difference in mortality between the two methods, the LOD strategy reduced direct medical costs by 23% [25]. This equivalence in outcomes, coupled with apparent cost-savings, resulted in the generation of consensus guidelines recommending that LOD after laparotomy for SCIAS be adopted as the standard of care [32]. However, neither LOD nor PRL arm included an open abdomen or negative peritoneal pressure therapy (NPPT). The mortality in this RCT of severe secondary peritonitis illustrates the devastating nature of this disease having a mortality of approximately 1/3 of all enrolled patients regardless of treatment allocation. This observed mortality rate calls out for ongoing examination of alternative approaches to manage SCIAS.

Pharmacologic approaches do not currently offer hope in SCIAS as studies of promising agents directed to combat post-infective inflammation have not shown evidence of significantly improved patient outcomes, and when suggested as having a role, have been incredibly expensive [33, 34]. Alternatively, OA is increasingly recommended as an option to control intraperitoneal contamination and to ameliorate the propagation of inflammatory bio-mediators in SCIAS [35–37].

The use of the OA for non-trauma general surgery is increasingly being reported in uncontrolled series as an option for patients with SCIAS [20, 28, 29, 38–40]. The use of the OA approach in SCIAS may increase drainage of residual infection, allow early identification and control of persistent infection, increase the removal of bio-mediator-rich peritoneal fluid, prophylaxis against the development of the abdominal compartment syndrome, and allow for the deferral of gastrointestinal anastomoses, with a potentially safer exit at the index operation [20]. However, compared to trauma patients, OA management for IAS patients has been reported to have a greater risk of complications, including enteroatmospheric fistula (EAF), intra-abdominal abscess, and a lower rate of primary fascial closure (i.e., fascia-to-fascia closure within the index hospitalization) [20, 21, 41] [42, 43]. Thus, there remains clinical equipoise in the regular use of the OA in SCIAS, with benefits and risks to adopting or avoiding its use.

### Metanalyses and randomized controlled studies of the open abdomen in trauma and sepsis

Although the use of Damage Control and an OA concept was once liberally embraced and assumed to be the ideal therapy for major trauma [44], sober critique has questioned the need for this approach and suggested that the treatment paradigm and actual intervention may be overused [45–47]. These concerns are germane when discussing non-trauma emergency surgical patients subjected to OA therapy as in IAS patients’ comorbidities are more common and more severe, closure rates are lower, and patients tend to be older and less able to withstand OA complications should they occur. Thus, it is important to have data unique to IAS patients to inform clinical decision-making.

Unfortunately, although case series on OA after non-trauma laparotomies have been reported, there are no contemporary RCTs. A recent meta-analysis on the use of Damage Control in perforated acute colonic diverticulitis [48], found no RCTs and ultimately the conclusions reverted back to opinions, the weakest level of Evidence in the World Society of Emergency Surgery Consensus Guidelines [49, 50]. In 2022, Cheng published a Cochrane Review on the use of negative pressure wound therapy for the non-trauma open abdomen and concluded that no recommendations could be made as there was no meaningful data [51]. Only one other RCT, conducted prior to 2006, has randomized 40 patients to a closed or open strategy, but the technique of OA management utilized then is inadequate according to current guidelines, as the NPPT apart from other aspects of OA management has evolved in technique and technology. This earlier RCT randomized patients with severe secondary peritonitis to an open or closed strategy after laparotomy, using a non-absorbable polypropylene (Marlex™) mesh in an interposed position between the open fascia, exposing the underlying bowel to the risk of enterocutaneous or enteroatmospheric fistula formation [52]. The study was stopped at the first interim analysis for futility. The risk of death was higher with the OA, but did not reach statistical significance, again leaving uncertainty as to how to treat patients [52]. Otherwise, there is no prospective randomized data and results other than that which will be collected in the COOL trial.

### Negative pressure peritoneal therapy (NPPT)

Newer non-commercial and commercial negative pressure peritoneal therapy (NPPT) systems are now available for OA and may reduce the risks of enterocutaneous fistula and facilitate enhanced delivery of negative peritoneal pressure to the peritoneal cavity [14, 32, 53]. In one of the largest contemporary OA databases, no difference in enterocutaneous fistula rates was noted related to the type of temporary abdominal closure dressing used [54]. However, there is a suggestion that more efficient peritoneal drainage may fundamentally impact the systemic complications of SCIAS. Animal studies [55] and in silica modeling of these animal studies [56] demonstrate that NNPT provides negative pressure and clearance of fluid throughout the peritoneum in contrast to simply leaving the fascia open with a temporary closure device. NPPT may reduce plasma bio-mediator levels compared to passive peritoneal drainage. Systemic inflammation (TNF-α, IL-1β, IL-6) in a single animal study was significantly reduced in the NPPT group and was associated with significant improvement in intestine, lung, kidney, and liver histopathology [55].

### Ugh—You want our advice? We don’t really know!

Many of the current investigators in the COOL trial also conducted the largest prospective randomized controlled trial addressing the question of differing NPPT in open abdomen management, the Intra-Peritoneal Vacuum Trial [35]. Patients were enrolled in the operating room after an attending surgeon made the decision that an OA approach was required in critically ill/injured patients. Serum bio-mediator levels were measured every 24 h in the initial post-laparotomy phase of critical care [35, 57]. Although standard systemic bio-mediator levels were not statistically different nor was peritoneal fluid drainage, the 90-day survival rate was higher in the NPPT group (P = 0.04) [35]. A valid critique of this trial was the heterogeneous mix of trauma and non-trauma patients [35]. A reasonable interpretation of this study’s results is that the study’s suggestion of a survivable benefit at minimum supports further investigation into therapeutic benefit in patients affected by severe SCIAS. In summary, great clinical equipoise remains as to whether the abdomen should be left open or closed after laparotomy in patients with SCIAS and warrants continuing to conduct the COOL Trial [38, 58].

### The globalization of COOL

The original intent of the COOL trial investigators was to examine an OA-NPPT technique that could be used anywhere [59]. The vision is to provide clinical operative guidance to surgeons with severe complicated abdominal sepsis as to whether they should close or not when the abdominal cavity is physically closeable. At the Inaugural Investigators COOL trial Meeting in Parma, Italy, the COOL trial Steering Committee endorsed the requirement to utilize an AbThera dressing (3 M, 3 M Center St. Paul, MN 55,144–1000). This decision was quite controversial and was fundamentally tied to financial trial support/sponsorship from the device manufacturer. It is important to clarify that apart from the use of the AbThera dressing, the sponsor was independent of the design or conduct of the study. The investigators assumed that the manufacturers of the AbThera would welcome the opportunity for an unbiased Global network of scientists to validate the efficacy of their proprietary device. This reflected the fact that the AbThera was only approved for use by the United States Food and Drug Administration, based on a so-called 510 K “loophole” that recognizes a substantial equivalence of the AbThera to 1976 predicate technologies, and not that the AbThera has ever been validated as better in any patient focused in rigorous human trials. Thus, the initial COOL Protocol required the use of a 3 M/Acelity AbThera dressing for any patient enrolled in the OA (intervention) arm of the Trial. This protocol stipulation was not without consequence as it precluded a “global” approach as many centers could not participate as the device was either not available and/or affordable.

### The potential to utilize other non-commercial negative peritoneal pressure abdominal dressings in the COOL Trial

On August 19, 2022, the 3 M Company, who had acquired the Acelity Corporation canceled support for the COOL trial [59]. The sponsorship contract for the trial did permit the Corporate Sponsor to cancel support any time without cause. While a major logistical problem for the COOL trial Investigators, an unanticipated benefit is the removal of the requirement for use of the specific AbThera dressing in the OA arm. The COOL trial was always designed to be pragmatic, and the original protocol upon which ethics approval was obtained was generic regarding OA and NPPT management. The intervention arm of the trial has simply required NPPT administered to an OA defined by the fascia not being formally closed following all four intraperitoneal quadrants washed until macroscopically clear [32]. Thus, any manner of mechanical devices [60, 61], or potential instillation therapies [62], are permitted adjuncts as long as the primary requirement for an open fascia with NPPT is met.

## Methods/Design

The current document is based upon the previously published COOL trial concise protocol [1], and outlines the evolution and lessons learned during the initial conduct of the COOL trial. Prompt resuscitation and the earliest possible appropriate antibiotic administration are critical for optimal outcomes in SCIAS. The COOL trial is pragmatic and will not stipulate specific protocols for such care, but emphasizes the importance of this for all patients whether not enrolled or enrolled into either arm of COOL.

### Objective/Aims

The aim of the COOL trial is to test the null hypothesis that there will be no difference in survival when an OA management strategy administering NPPT is utilized compared to a primary fascial closure strategy in patients with SCIAS. The study is designed as a prospective, single-blinded, multicenter, international RCT. A SPIRIT Diagram overview of the trial is presented in Table 1. The complete protocol as well as a rich library of study-related documentation is available at www.coolstudy.ca.

**Table 1:**
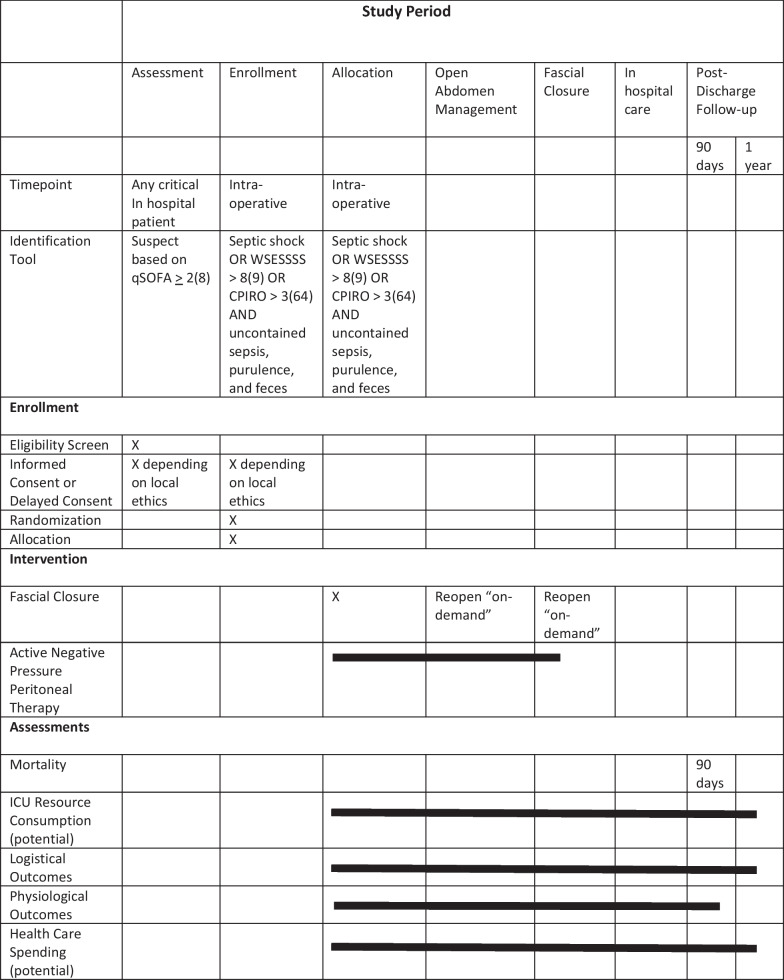
SPRIT Diagram describing schedule of enrollment, interventions, and assessments

1. Singer et al. [8]

2. Sartelli et al. [9]

3. Posadas-Calleja et al. [64]

### Setting

The COOL trial is being conducted in operating rooms around the world where critically ill patients with SCIAS undergo source control laparotomy. The lead study center is the Foothills Medical Centre, a Quaternary Care Academic Medical Centre located in Calgary, Alberta, Canada. To date, thirteen hospitals on four continents have enrolled patients in the COOL trial, although more centers are open for recruitment.

### Inclusion/exclusion criteria

**Fig. 1:**
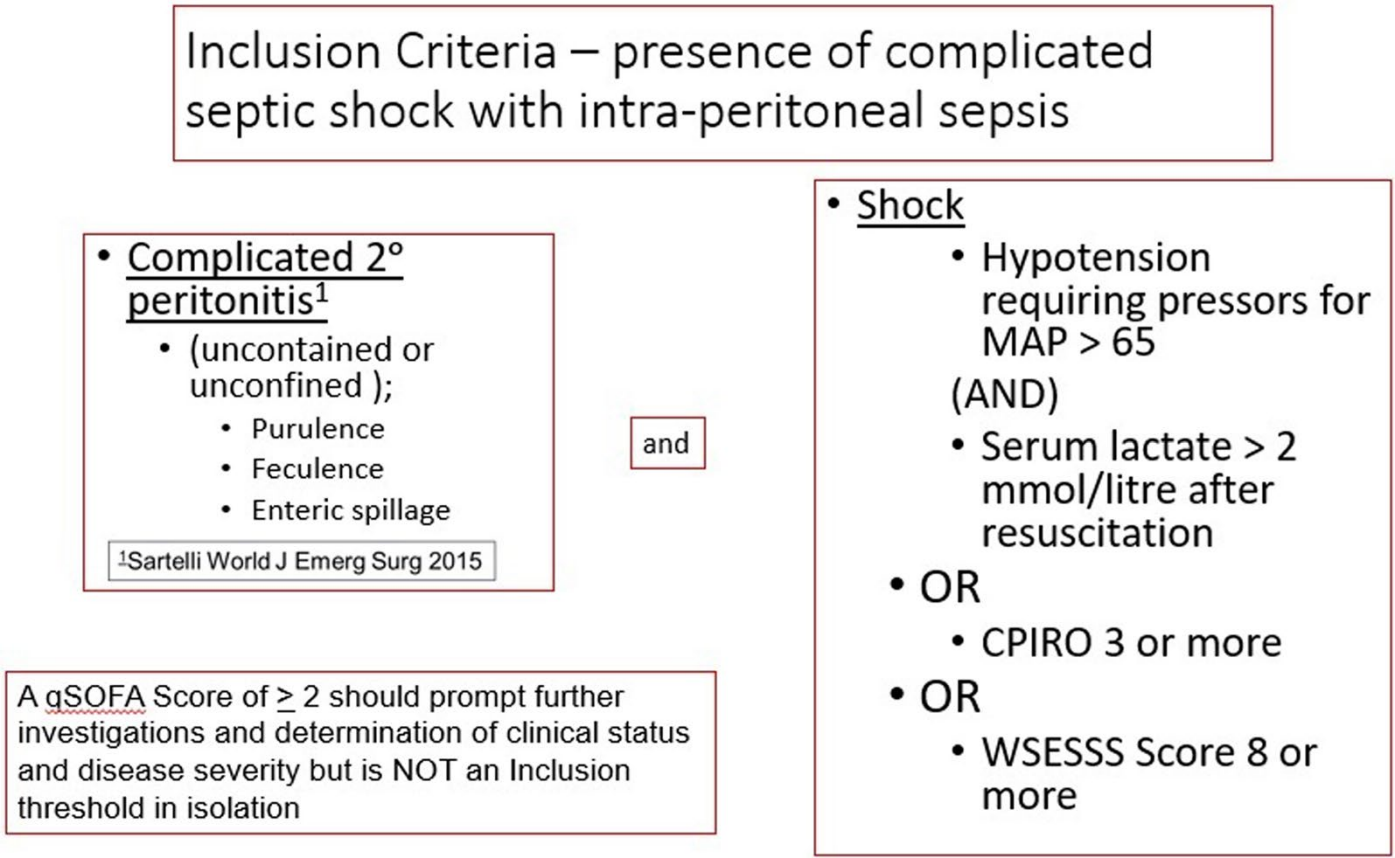
Inclusion criteria for COOL

Potential patients will first be identified in the emergency departments, in-patient ward, and critical care units of the participating centers. Eligibility can only be completely determined after the abdomen is explored in the operating room during the conduct of a laparotomy for source control. Patients will be eligible for inclusion if they have SCIAS, as operationally defined by the COOL trial (Fig. 1).

The inclusion criteria are conceptually a two-part assessment to ascertain if patients clearly fulfill the definition of both severe and complicated IAS (SCIAS) while undergoing source control laparotomy. Thus, during the laparotomy it will become apparent to the operating surgical team that peritonitis is complicated, which will be reproducibly demonstrated by uncontained or unconfined purulent, feculent, or enteric spillage. In addition to being complicated, the inclusion criteria require that patients have severe IAS. For the purpose of the COOL trial, severe will be defined by any of: septic shock as defined by Sepsis 3 Consensus Guidelines [8], a World Society of Emergency Surgery Sepsis Severity Score > 8 [9], or a Calgary Predisposition-Infection-Response-Organ Dysfunction Score > 3 [63]. An elaborated explanation of the thought processes and identification attributes of these criteria modeled on a trial population of SCIAS patients was previously published by the COOL trial Investigators [64].

The exclusion criteria for the COOL trial include: a) pregnancy, b) perceived physical inability to physically close the fascia primarily without undue tension or concerns for inducing severe IAH/ACS, c) intra-operatively determined absolute or relative requirement for “damage control” laparotomy including intraperitoneal packing or non-anatomic postsurgical anatomy (i.e., surgically placed permanent packing or bowel that the operating surgeon believes must be left in discontinuity after resection), d) the patient is expected to die shortly after operation because of their condition in the operating room and there is no intention of providing ongoing care (i.e., the treating team wishes to close the abdomen to leave the operating room with the sole intention of withdrawing aggressive measures and providing only “comfort care” in the ICU; an example of where this could occur would be complete transmural midgut ischemia/necrosis), e) laparoscopic surgery (no laparotomy), f) pancreatitis as the source of peritonitis, g) acute superior mesenteric artery occlusion as the primary pathology, h) co-enrollment in another investigational study, i) peritoneal carcinomatosis, j) traumatic injury within 24 h of the development of SCIAS, k) age < 18, or l) uncontrolled bleeding. It will be important for surgeons considering recruiting a patient to recognize before enrolling and randomizing a patient that fascial closure is not possible, as recognizing this after allocation to closure will constitute a protocol violation.

In current practice, it is likely that the most common reason for non-eligibility will be a surgeon-based decision to resect a hollow viscus and due to the perceived critical nature of the patient decide not to re-anastomose the bowel but to instead perform damage control and return the bowel ends into the peritoneal cavity without a diverting stoma. As this is an absolute indication for a future reoperation these patients will be ineligible for randomization.

### Randomization

**Fig. 2:**
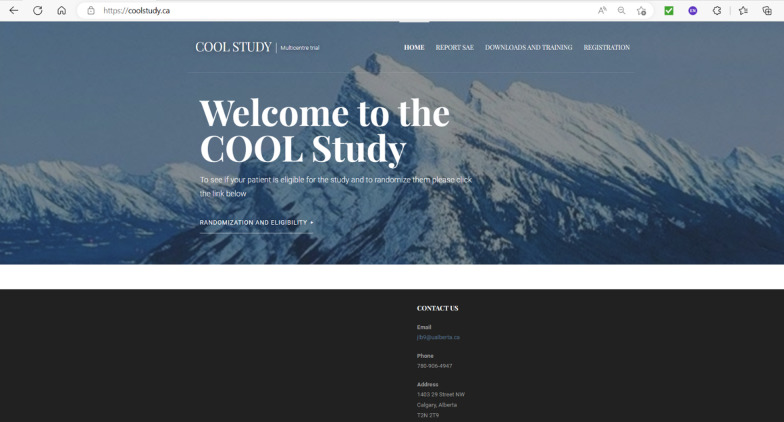
COOL trial enrollment site

Treatment arm allocation is randomly allocated from a central, password-protected, randomization Web site (www.coolstudy.ca) (Fig. 2). This can be done from any computer or smartphone and accessing the enrollment site for randomization need not be conducted by the attending surgeon. The ability to enroll a patient can be accessed with a password by any member of the surgical/anesthesia/critical care medicine/nursing team. When an appropriate patient is recognized, the research Web site will be accessed, simple identifiers of the patient will be entered, and treatment allocation (CLOSED with fascial closure or OPEN with an NPPT TAC dressing being applied) associated with this entry will be generated. To ensure the close balance of the numbers in each of the two treatment groups, permuted block randomization by site will be used. If the operating team is uncertain regarding the potential stratified severity according to either the WSESSSS or CPIRO methods, online decision support software simplifies these calculations regarding any potential enrollment.

### Primary closure—CLOSED allocation (Control arm)

This strategy consists of primary closure of the fascia using any technique or suture material as chosen by the attending surgical team. Closure of the skin and the method for preventing surgical site infections is left to the discretion of the attending surgeon. There is no expectation for relaparotomy. Postoperative diagnostic imaging and all other aspects of postoperative care including any decision to perform a relaparotomy shall be at the discretion of the treating critical care/surgical teams. A decision to perform a relaparotomy will constitute a study outcome. If at any subsequent laparotomy the attending and responsible surgeon selects an open abdominal strategy (crossover to the intervention arm), the outcomes will be analyzed based on the intention-to-treat allocation at the time of original enrollment. Any application of any wound suction or negative pressure device to the soft tissue above a closed fascia is permitted within the control arm (closed abdomen).

### Open Abdomen with Negative Pressure Peritoneal Therapy—OPEN allocation (Intervention arm)

Once the patient has been allocated to an OA, the trial protocol does not mandate the interval until fascial closure although the intention is that closure will occur expeditiously once clinically determined safe by the treating surgeon. The COOL trial protocol does not mandate any length of OA therapy, although the principle of the earliest safe formal closure is expected. The time that the temporary abdominal closure dressing will be left in place, will be left to the discretion of the attending surgeon, but typical practice guidelines mandate either formal abdominal closure or dressing changes at 24–72 h if formal abdominal closure cannot be completed [49]. For both arms of the trial, it will be expected that attending surgeons are involved in either the direct supervision and/or intra-operative participation with either facial closure or temporary abdominal closure. The trial is considered pragmatic in allowing a variety of techniques as long as NPPT is being administered to an OA defined by the fascia not being formally closed and that all four intraperitoneal quadrants have been washed until macroscopically clear [32]. A suitable NPPT dressing must provide a complete viscero-protective layer, a means of the controlled egress of intraperitoneal fluid, and negative pressure within the peritoneal cavity. Thus, any manner of mechanical traction devices [60, 61, 65], or potential instillation therapies [62], will be permitted adjuncts as long as the primary requirement for an open fascia with NPPT is met. Terminology related to closure strategies and explicit methods used will be consistent with the terminology detailed in both the World Society of the Abdominal Compartment Syndromes most recent consensus definitions and practice management guidelines and those of the Open Abdomen Advisory Panel [32, 66, 67]. When the COOL trial was initiated, the commercial AbThera dressing was mandated, but this requirement was amended on August 2022 following 3 M’s termination of the contract and sponsorship. Thus, other centers from countries that choose to use any other negative pressure dressing will be permitted; the type of NPPT will be considered in a subgroup analysis.

### Outcomes

The primary outcome will be survival at 90 days from enrollment. Recent systematic reviews reveal higher mortality for both sepsis and septic shock populations examining 90- versus 30-day mortality rates and thus the 90 days of time frame was felt to provide a more comprehensive understanding of SCIAS mortality [68]. Secondary outcomes will be logistical and physiologic (Table 2). Logistical outcomes will include Days Free Of (DFO); ICU, ventilation, renal replacement therapy, and hospital at 90 days from the Index Laparotomy. The physiological secondary outcomes will include a change in APACHE II, SOFA, and ARDS scores after laparotomy. The COOL trial inclusion criteria concerning intraperitoneal contamination will be recorded, and the index source control laparotomy and every subsequent laparotomy will be graded according to the OA classification system from the 2013 World Society of Abdominal Compartment Syndrome (WSACS) grading scale for OA [32, 69, 70]. Surgical complications occurring after the index laparotomy will be graded according to Clavien-Dindo (Grade I = any deviation from normal postoperative course, including wound infections opened at the bedside but not treated with antibiotics; Grade II = requiring pharmacological treatment, e.g., antibiotic treatment, blood transfusion or parenteral nutrition; Grade IIIa = requiring surgical, endoscopic or radiologic intervention without general anesthesia and IIIb under general anesthesia; Grade IVa = life-threatening complication requiring IC/ICU management with single organ dysfunction and IVb with multiorgan dysfunction; Grade V = death of patient) [71, 72].

**Table 2:** Overview of study outcomes

	Indicator	Timeline
Primary outcome	Mortality	90 days
Secondary outcomes		
Logistical	Days free of ICU	30 days
	Days free of ventilation	30 days
	Days free of RRT^a^	30 days
	Days free of hospital	30 days
Physiological	APACHE II^b^ scoresup to	30 days^c^
	SOFA^d^ scoresup to	30 days^c^
	Pa02/Fi02^e^ ratiosup to	30 days^c^
	ARDS^f^ scoresup to	30 days^c^
Safety	enterocutaneous fistula	30 days
	ACS^g^ and/or severe IAH^h^	30 days
	Intra-abdominal abscess	30 days
	Need for reoperation	30 days

^a^RRT = Renal Replacement Therapy

^b^Acute Physiology and Chronic Health Evaluation Score

^c^Measured daily using the worst value of that day

^d^SOFA = Sequential organ Failure Assessment

^e^Pa0_2_/Fi0_2_ = Partial pressure of oxygen over inspired fraction of oxygen

^f^ARDS = Acute Respiratory Distress Syndrome

^g^ACS = Abdominal Compartment Syndrome

^h^IAH = Intrabdominal Hypertension

**Fig. 3:**
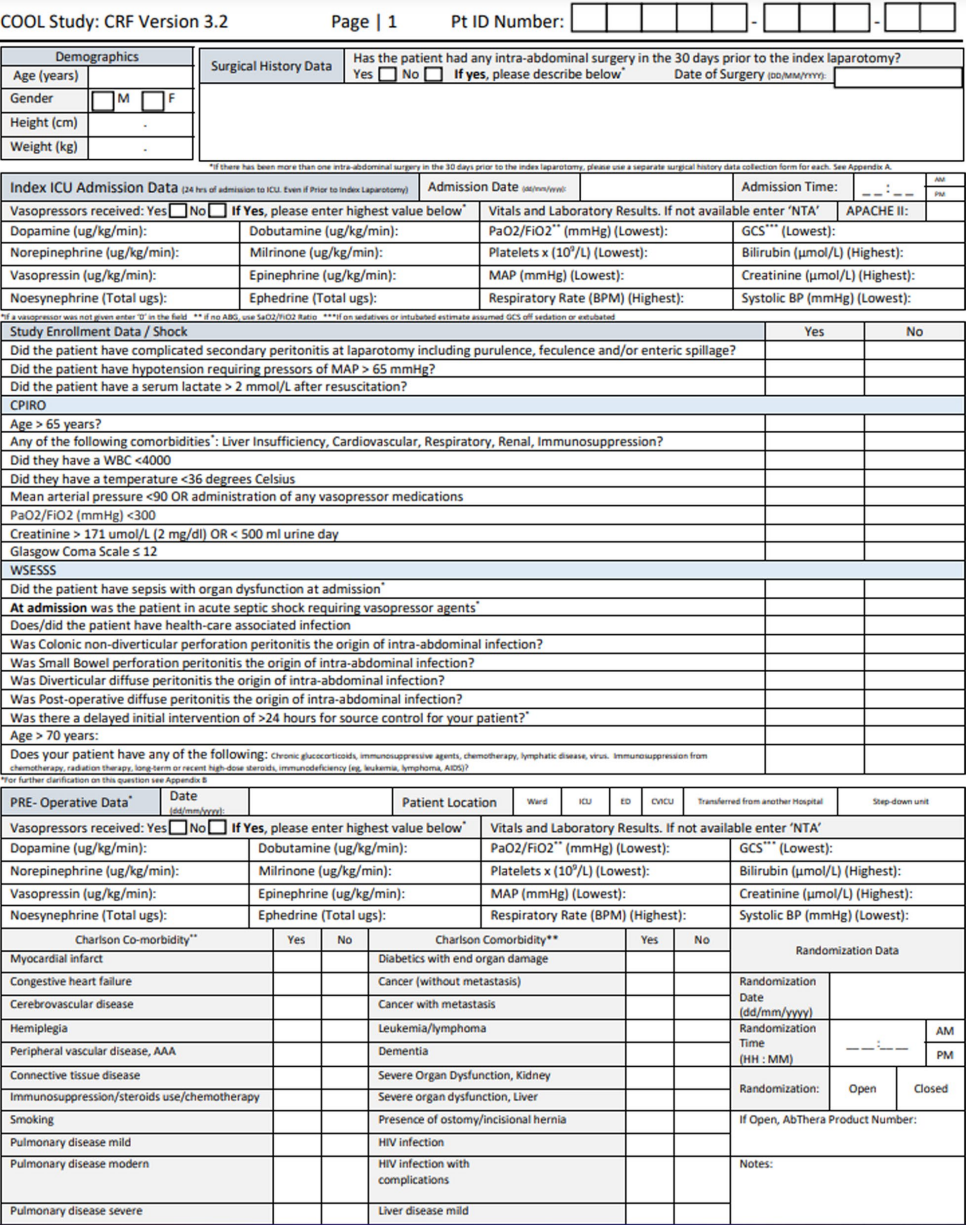
COOL study case report form. The Case Report Form is a extensive document that can be accessed online at Study Documents – COOL Study, but Investigators are encouraged to complete the form on-line where it will be securely entered into the University of Calgary REDCap (R esearch E lectronic D ata Cap ture) database.

All data are entered into a secure web application for building and managing online surveys and databases (REDCap) maintained by the University of Calgary. While the COOL Trial Case Report form is available in paper format (Fig. 3), investigators are encouraged to submit data directly into the online format securely hosted in REDCap (Research Electronic Data Capture). The Case Report Form (CRF) was also recently simplified to become more pragmatic in anticipation of an increasingly global participation with less dedicated research administration. Although an immensely detailed and exhaustive COOL trial database would facilitate future “spin-off” studies, this should not be constructed at the expense of exhausting global collaborators dedicated to participate, but with limited research resources.

### The Evolution of COOL over COVID and other World Crises

The initial protocol for the COOL trial envisioned multiple nested studies examining all aspects of OA management, of which an adequately powered trial of mortality was the centerpiece [1]. Thus, any hospital providing emergency surgical services with intensive care support can participate if they are committed to recruit and randomize patients with SCIAS fulfilling the eligibility criteria during source control laparotomies. Contributing toward this main outcome will require only collection of the clinical outcome data. Prospective sub-studies that were envisioned to augment this main goal included COOL-Max (Bio-mediators), COOL-Mic (Microbiology), COOL-Cells (cellular defense mechanism), and COOL-Costs (economics). After the initiation of the clinical COOL trial, it became apparent that realistic operational demands and economic limitations precluded the conduct of these sub-studies, although a retrospective COOL trial economic analysis of open versus closed treatment is still a practical future analysis [73]. Thus, the dedicated focus of the current COOL trial efforts is completing the clinical outcome analysis powered on mortality.

### Sample size calculations

The COOL trial is overall powered to detect a significant difference in the primary outcome, 90-day survival. Although imperfect, the preceding Intra-Peritoneal Vacuum Trial study revealed an Intention-to-treat 90-day mortality of 21.7% in the ABThera group versus 50.0% in the Barker’s vacuum pack group [HR, 0.32; 95% confidence interval (CI), 0.11– 0.93; P = 0.04] [74]. This 30% reduction in mortality was considered too dramatic to be practically replicated and a much more conservative effective 10% reduction in mortality was chosen. Thus, given a mortality rate of 33% in the general population of those with severe intra-abdominal sepsis, and considering a power of 80% and an alpha of 0.05, the number needed to recruit was calculated as 275 patients in each arm.

### Statistical analyses

The effectiveness of randomization will be displayed through a detailed presentation of patient demographic characteristics as outlined in Table 3. The analysis of the primary outcome, mortality, will be on an intention-to-treat basis related to the allocation of initial intra-operative therapy. There will be a planned subgroup analysis of the mortality stratifying patients into those with and without the presence of septic shock (defined as Sepsis-3 Consensus Guidelines) during the first 48 h after onset of peritonitis (if known and 24 h before and 24 h after 1st laparotomy if not known). There will also be a planned subgroup analysis looking for any difference in outcomes within the intervention arm of the study between patients managed with the AbThera commercial dressing and any other NPPT dressing.

**Table 3:** Baseline demographic characteristics of the study patients

Male/female
Age, median (IQR^a^), years
Septic shock^b^
World Society of Emergency Surgery Sepsis Severity Score^c^
Calgary PIRO Score^d^
GCS^e^, median (IQR)
APACHE II^f^, median (IQR)
Arterial pH, mean (95% CI)
Base deficit, median (IQR)
Lactate, median (IQR)
INR^g^, median (IQR)
Temperature, mean (95% CI)
APACHE II score^f^, mean ± SDd
SOFA score^h^, mean ± SDe
Charlson Comorbidity Index score^i^, median (IQR)
Worst physiologic measurements prior to randomization, median (IQR)
Systolic blood pressure, mmHg
Temperature (injured patients), °C
Temperature (sepsis patients), °C
pH
Lactate, mmol/L
Base deficit, mmol/L
INR
Fluid administration prior to randomization, median (IQR)
PRBC^j^, units
FFP^k^, units
PRBC/FFP ratio
Crystalloid, L
Patient location prior to OR admission—no. (%)
Emergency Department
Hospital ward
Intensive Care Unit
Vasopressors required prior to randomization—no. (%)
Hours from sepsis diagnosis to laparotomy, median (IQR)
If allocated to OPEN
Type of Negative pressure peritoneal therapy (NPPT) temporary abdominal closure (TAC) device

^a^IQR, interquartile range

^b^septic shock as defined by SESPS-3 Guidelines[8]

^c^WSESSS [9]

^d^CPIRO [63]

^e^GCS—Glasgow coma score

^f^Acute Physiology And Chronic Health Evaluation II

^g^INR—international normalized ratio

^h^SOFA—Sequential Assessment of Organ Failure [137]

^i^Charlson Comorbidity Index(138)

^j^PRBC—packed red blood cells

^k^FFP—fresh frozen plasma

There will be a single interim analysis planned after the recruitment of 275 patients, which will analyze the difference in 90-day mortality between allocated therapies. The COOL trial Investigators appreciate the general reluctance to stop randomized trials early due to benefit, due to the frequent over-estimating of treatment effects [75–77]. Despite this, if a profoundly significant difference is found (p < 0.01) the trial will be stopped, otherwise it will continue to full recruitment.

### Ethical concerns

There is clinical equipoise concerning the operative management of SCIAS. Thus, the COOL trial Investigators feel a moral imperative to conduct this research to provide the best evidence to counsel bedside critical care physicians and surgeons [78]. The COOL trial is currently approved by the Conjoint Health Research Ethics Board of the University of Calgary (REB-16-1588) to proceed with a delayed consent process given the time-sensitive critical nature of decision-making. Research ethics will vary throughout the world and it is anticipated that various local policies concerning community consent, waiver of consent, or informed consent of significant patient proxies may be considered. All participating Institutions will thus be required to obtain Ethical Approval appropriate and applicable to their Institutions.

Research in critically ill incapacitated patients is important to advance care. Conducting research among SCIAS is complicated due to the severity of illness, need for emergent interventions, diagnostic criteria confirmed only at laparotomy, and obtundation from anesthesia. In other circumstances involving critically ill patients, clinical experts have worked closely with ethicists to apply principles that balance the rights of patients while simultaneously permitting inclusion in research. COOL Investigators have collaborated with both current and past Chairs of REB’s to review and interpret the science and ethics for surgical investigators globally [79, 80]. The ultimate goal is to balance respect for patient participants and to permit participation with a reasonable opportunity for improved outcome and minimal risk of harm.

## Discussion

Randomized surgical trials, especially those not supported by industry are notoriously few, hard to complete, and increasingly poorly supported by traditional granting agencies [81–83]. Yet these trials are desperately required. In general, the overall quality of surgical research can be criticized as being grossly inadequate despite being the purported basis of surgeons making evidence-informed decisions with an impact which may affect a patient’s outcome including death or being permanently impaired [79, 80, 84, 85]. One famous commentary compared surgical research to “comic opera” [86], lamenting the reliance on retrospective case series as a methodology, and another referred to the typical retrospective case series (that constitute the near totality of research concerning SCIAS) as “research waste” [84]. Unfortunately, retrospective case series predominate, potentially because they are vastly easier to conduct, are free of regulatory hurdles that accompany conducting an RCT, are publishable in journals and offer career advancement to investigators. However, why surgical RCTs are so few may also relate to fundamental differences in the regulatory approval process between medicines and medical devices. Whereas the level of confidence in pharmaceutical safety has risen substantially since the Thalidomide debacle [87], comparable changes in the safety bar to approve medical devices are less well developed. Thus, RCTs are often not required by device manufacturers or regulators to allow market entry [84], and thus research funding for devices demonstrating a beneficial effect on outcome is often lacking.

Nonetheless, the COOL trial has been designed to answer a critical clinical question that faces clinicians worldwide on a daily basis for which there is important clinical equipoise and potential severe consequences for patients in regards to outcomes [38, 58]. Thus, this question has been identified as one requiring urgent study [49]. The COOL trial has continued to be supported by not-for-profit Scientific Organizations with a vested interest in the best care of the critically ill patient including the Abdominal Compartment Society and the World Society of Emergency Surgery. The trial design and vision follow directly from the preceding single-center study of differing modalities of NPPT conducted at the Foothills Medical Centre [57, 74]. When the Intra-Peritoneal Vacuum Trial investigators considered following up the pilot study and enrolling more patients in a multicenter fashion, it became apparent after peer-to-peer discussion that any differing effectiveness of NPPT techniques was not the most relevant question concerning the OA [88]. With an evolution in resuscitation practices involving balanced resuscitation, more and more trauma patients who previously become so edematous they required OA therapy, are no longer being over-resuscitated with crystalloids, and can be primarily closed [89–91]. This change in at least the trauma care paradigm has justified questions regarding the whole premise of damage control surgery for trauma [92], and IAS [45].

As over-resuscitation becomes less common [93, 94], it is intuitive that there will be more abdomens in non-trauma IAS patients which may be technically closed without inducing intra-abdominal hypertension (IAH). However, although these abdomens *may* be closed, *should* they be closed? As has been recently emphasized, there are profound differences in the basic science of sepsis and traumatic injury [95], with the previously unifying concepts of non-infectious Systemic Inflammatory Response Syndrome (SIRS) being effectively discarded as a clinically helpful construct [8, 96, 97]. The one nebulous, poorly defined “holy grail” of the optimal management of SCIAS is adequate “source control.” It is suggested that even if an abdomen can be physically closed that there may be an advantage to leaving it open for a brief period to allow better drainage of intraperitoneal contamination, a concept that is supported by animal data suggesting the ability of NPPT to mitigate the elaboration of the inflammatory bio-mediator cascade [55, 56, 98], although this has not been demonstrated in humans [74].

### The peritoneal cavity as a reservoir for systemic inflammation

There is a complex relationship between pressure, ischemia, and inflammation within the peritoneal cavity [14, 16]. Independently the damaged gut seems to act as a continued source of inflammation propagating SIRS and potentiating MODS [99–103]. Basic, predominantly animal laboratory research, from the last decade suggests an exciting potential. Visceral ischemia characteristically generates multiple immunological mediators with the pro-inflammatory cytokines tumor necrosis factor-alpha (TNF-α), and interleukin six (IL-6), as well as inhibitive cytokines such as interleukin ten (IL-10) [104–107]. Postoperative complications are associated with increasing levels of systemic IL-6, and peritoneal TNF-α [106, 108]. Jansson and colleagues thus postulated that peritoneal cytokines in humans respond more extensively compared to systemic cytokine, and that a normal postoperative course is characterized by decreasing levels of peritoneal cytokines based on studies of both elective and emergency surgery [109]. Overall, the peritoneal cytokine response is much higher than the systemic response in peritonitis [107, 110–112]. Hendriks and colleagues demonstrated that peritoneal cytokine levels (especially IL-6, TNF-α [113], and IL-10) were dramatically different in rats who either survived or succumbed to an IAS model in the 24 h after cytokine determination [110]. Finally, recent work suggests that blood filters designed to hemofiltrate blood endotoxins and cytokines may improve hemodynamics, organ dysfunction and even mortality in the critically ill [114–117].

The biologic rationale for COOL is that if safe, removing intraperitoneal bio-mediators may mitigate their local effects and prevent their being absorbed systemically. Although early work suggested a benefit of simple continuous peritoneal lavage after either gross peritoneal contamination in secondary peritonitis or in the setting of necrotizing pancreatitis [118, 119], subsequent studies could not confirm a benefit [120–122]. Studies using hemofiltration to remove inflammatory mediators from the blood have been associated with reduced elevations of inflammatory cytokines (as assessed by blood IL-6 levels), early improvements in hemodynamic state and decreased lactate levels [123–125]. However, it has not yet been demonstrated that extracorporeal filtration of inflammatory mediators improves clinical outcomes [126, 127]. One possible explanation for this is that after the mediators have left the peritoneal cavity and become systemic the “horse is out of the barn.”

NPPT therapy may be a more direct, earlier, and focused solution to this complicated problem, and one that will be complementary to the other benefits of OA. Whether improved postoperative courses can be obtained through this relatively simpler approach of actively removing peritoneal cytokines with a more efficient and comprehensive VAC therapy in humans is therefore part of the biologic rationale of the COOL trial.

Another potential benefit of NPPT after severe infection may be the attendant decompression of the abdominal compartment and prevention of even modest IAH. Patients with intra-abdominal infections are at risk of elevated IAP both because of the primary intraperitoneal disease, and as a consequence of the use of large-volume crystalloid resuscitation often used to maintain organ perfusion [128–130]. Recent studies have demonstrated a high prevalence of IAH following aggressive volume resuscitation of septic patients. IAH is present in as many as 80% of septic medical and surgical ICU patients [131, 132]. Reintam also reported that septic patients with IAH had a 50% mortality rate compared to 19% without IAH, making IAH a significant marker for an increased risk of death [133]. Within the lead COOL Institution rates of IAH were over 87% of septic ICU patients and further 61% of these patients had severe IAH at levels commensurate with ACS, despite the fact that IAP was only measured in 10% of the patients in whom guidelines recommend monitoring [134]. Although direct translation to humans is uncertain, even modest degrees of IAH (often clinically ignored) have been found to have profound far-reaching effects on propagating multiple organ failure in animals with ischemia/intraperitoneal infections [135–137].

### COOL trial recruitment

Like many, especially investigator-initiated randomized trials, recruitment has lagged behind original predictions for the COOL trial. Poor participant recruitment is the most frequent cause for premature discontinuation of randomized clinical trials [138, 139]. The COOL trial has competed with the COVID pandemic as a novel challenge apart from other established causes for poor trial enrollment such as inadequate funding, a narrow (but necessary) eligibility criteria, and a de-emphasis of research priorities even in University hospitals [138]. The financial burden of Clinical Trial Insurance has been a particularly challenging burden to the COOL trial. The difficulty in financing was made worse by 3 M canceling its contract to support the COOL trial. However, recruitment is measured against an arbitrary prediction, so the true adequacy of recruitment will only be assessable when the outcome data is formally analyzed. Although this is not planned until 275 patients have been recruited, it is relevant that at this time COOL is nearly twice as large as the most relevant RCT previously reported [52]. Thus, as new centers are added (as they have been monthly) the COOL trial will continue and should be successful in meeting its enrollment goals.

## Conclusions

The COOL trial is designed to examine if a mortality difference exists in this highly lethal and morbid condition, and to ensure critically ill patients are receiving the best care possible and not being harmed by inappropriate interventions or devices based on opinion only. The COOL trial Investigators now welcome truly global participation for all interested surgical scientists and their supporters.

## Data Availability

All results and data from the COOL trial will be available from Dr. Andrew Kirkpatrick (andrew.kirkpatrick@albertahealthservices.ca) on reasonable request, as well as from the Study Web site (www.coolstudy.ca).

## Abbreviations

COOL: Closed Or Open after Laparotomy trial (https://clinicaltrials.gov/ct2/show/NCT03163095)
SCIAS: Severe complicated intra-abdominal sepsis
OA: Open abdomen
NPPT: Negative pressure peritoneal therapy
IAS: Intra-abdominal sepsis
RCT: Randomized Controlled Trial
LOD: Laparotomy on demand
PRL: Planned relaparotomy
EAF: Enteroatmospheric fistula
CPIRO: Calgary Predisposition, Infection, Response, and Organ Dysfunction
APACHE: Acute Physiology and Chronic Health Evaluation
SOFA: Sequential Organ Failure Assessment
ARDS: Acute respiratory distress syndrome
MODS: Multiple Organ Dysfunction Score
SIRS: Systemic inflammatory response syndrome
qSOFA: Quick SOFA score
WSESSSS: World Society of Emergency Surgery Sepsis Severity Score
APC: Activated protein C
mtDNA: Mitochondrial DNA
C5a: Complement factor 5 activated
C3a: Complement factor 3 activated
REDCap: (Research Electronic Data Capture) database
REB: Research Ethics Board
TAC: Temporary abdominal closure
IAH: Intra-abdominal hypertension
IAP: Intra-abdominal pressure
ACS: Abdominal compartment syndrome
